# Chemical predator signals induce metabolic suppression in rock goby (*Gobius paganellus*)

**DOI:** 10.1371/journal.pone.0209286

**Published:** 2018-12-17

**Authors:** Nina Paul, Sara C. Novais, Marco F. L. Lemos, Andreas Kunzmann

**Affiliations:** 1 Leibniz Centre for Tropical Marine Research (ZMT), Bremen, Germany; 2 MARE–Marine and Environmental Sciences Centre, ESTM, Instituto Politécnico de Leiria, Peniche, Portugal; Uppsala Universitet, SWEDEN

## Abstract

In nature, a multitude of both abiotic and biotic stressors influence organisms with regard to their overall fitness. Stress responses that finally impair normal biological functions may ultimately result in consequences for whole populations. This study focused on the metabolic response of the intertidal rock pool fish *Gobius paganellus* towards simulated predation risk. Individuals were exposed to a mixture of skin extracts from conspecifics and chemical alarm cues from a top predator, *Octopus vulgaris*. Oxygen consumption rates of single fish were measured to establish standard (SMR) and routine metabolic rates (RMR) of *G*. *paganellus*, and to address the direct response towards simulated predation risk, compared to handling and light stress. The SMR of *G*. *paganellus* (0.0301 ± 0.0081 mg O_2_ h^-1^ g^-1^ WW) was significantly lower than the RMR (0.0409 ± 0.0078 mg O_2_ h^-1^ g^-1^ WW). In contrast to increased respiration due to handling and light stress, the exposure to chemical predation cues induced a significant reduction in oxygen consumption rates (0.0297 ± 0.0077 mg O_2_ h^-1^ g^-1^ WW). This metabolic suppression was interpreted as a result of the stereotypic freezing behaviour as antipredator response of gobiid fish. Results underline the importance of biotic interactions in environmental stress assessments and predation as a biotic factor that will provide more realistic scenarios when addressing stress impacts in tidal rock pool organisms.

## Introduction

In an ecosystem, both abiotic and biotic (e.g., intra- and/or interspecific competition for space, food, mating partners) stressors may affect organisms and have impacts on somatic growth, reproduction, and disease resistance, which might ultimately result in consequences for whole populations (e.g., [1,2,3,4,5,6,7]). Under abiotic stress, organisms are often more vulnerable to biotic stressors [8]. On the other hand, biotic interactions can affect the individuals’ ability to cope with abiotic stress [9,10]. One of the most prominent biotic interactions is the predator/prey relation. From the prey’s point of view, proper predation risk assessment is of life-saving importance (reviewed by [11]). Fish that are able to avoid high-risk areas [12] can significantly increase their fitness, as Brown et al. (1995) report for fathead minnows (*Pimephales promelas*) [13]. These findings are in line with the concept of the threat-sensitivity hypothesis introduced by Helfman (1989) that predicts an increase in anti-predator behaviour when predation risk increases [11].

Between terrestrial and aquatic environments, there are differences in the effectiveness of senses used for the predation risk assessment, as auditory senses are often ineffective for aquatic organisms. Numerous studies used visual cues by presenting real-life predators or predator models to the target organisms (e.g., [11,14,15]). However, visual senses are often limited [16], e.g., in turbid water or highly complex habitats like rocky shores and coral reef structures. Therefore, chemical cues in particular are important in the aquatic environments (e.g., reviewed by [12,16,17,18]).

There are different groups of chemical cues that can be released and/or detected by aquatic organisms (reviewed by [18]). Predators can be identified due to a specific odour, so-called kairomones [16,18]. Kairomones are defined as “chemicals released by one species (predator), received by a second species (prey), that is adaptively favourable to the second species but not the first” [18]. Apart from the predator’s odour, prey also respond to dietary cues from digestion processes released by the predator after feeding on prey conspecifics [18]. Prey specimens can also actively release chemical pre-attack alarm signals to warn conspecifics when disturbed by a predator. In addition, conspecifics’ damaged-released alarm cues resulting from injured epidermal tissue after attack can passively warn conspecifics from actively foraging predators [18,19]. A mixture of different chemical stimuli containing both kairomones (indicating a predator being present in the closer environment) and alarm substances in skin extracts of conspecifics (simulating the predator actively feeding on their conspecifics) have thus the potential to increase the level of threat for prey organisms (e.g., [11,14]).

While some organisms are able to avoid predators by fleeing, others are faced with limited behavioural options [20]. In contrast to pelagic species, organisms that inhabit rocky intertidal zones are often limited in space, especially during low tide. The rock goby (*Gobius paganellus*, Linnaeus, 1758) is such a demersal, amphidromous fish belonging to the family Gobiidae, that can be found in intertidal waters and rocky shores at a depth range of 0–15 metres [21]. Its wide distribution from temperate towards sub-tropical regions (60°N– 12°N, 32°W– 42°E [21]) but sedentary way of life makes *G*. *paganellus* a suitable model organism for the study of climate change effects in intertidal habitats. During ebb tide, specimens are frequently found in shallow rock pools and below gravels high on the shoreline, which makes them prone to abiotic and biotic stressors. Recent studies investigated thermal stress responses of *G*. *paganellus* using biomarkers related with oxidative stress [22,23,24]. However, research on metabolic responses towards biotic stressors in *G*. *paganellus*, namely predation stress, and eventual implications in these organism’s populations is scarce.

The main goal of this study was to investigate the metabolic response of *G*. *paganellus* to chemical predator alarm cues, assuring a functioning predation stress simulation, using a mixture of kairomones, dietary cues, and skin extracts from conspecifics. Several studies investigated behavioural responses of gobiid fishes towards predation threat (e.g., [25,26,27,28,29,30]). Throughout all investigations, the fishes have responded with reduced activity, namely “freezing” behaviour [26], both in aquarium-based experiments [26,27,28,30] and field observations [29]. However, behavioural observations are difficult to interpret [20] and metabolic impairments are hardly detectable [20,31]. The measurement of oxygen consumption (OC) rates is therefore a common tool in stress assessments and a preferred way to estimate the metabolism of organisms (reviewed by [32]), which has successfully been used in analysing metabolic responses to predatory threat in aquatic organisms (e.g., [33,34,35]). The standard metabolic rate (SMR) is defined as the “minimum metabolic rate of survival” [36], and routine metabolic rate (RMR) is defined as the oxygen consumption of undisturbed, post-absorptive fish, which is higher than SMR. The metabolic responses towards stressors can finally be evaluated by comparing the OC rates during stress with determined SMR and RMR. Both SMR and RMR were successfully used for instance in determining metabolic rate relationships of intertidal mud crabs in absence and presence of predation threat [35]. To our knowledge, however, this is the first time assessing the metabolic response of *G*. *paganellus* towards chemically induced predation stress, while comparing it with handling stress [37,38,39] and visual disturbance due to abrupt change from dark to light. All stress responses were finally evaluated with respect to the SMR and RMR of *G*. *paganellus*. Based on observations of previous studies mentioned above, the following hypotheses were tested: (1) handling stress and visual disturbance significantly increase the OC rates compared to RMR; (2) OC rates at predation stress are significantly lower than OC rates at handling stress and visual disturbance; (3) OC rates at predation stress are below RMR, providing metabolic validation of the stereotypic freezing behaviour reported in several previous studies.

## Material and methods

In this study, oxygen consumption (OC) rates of single fish were measured to establish standard (SMR) and routine (RMR) metabolic rate of *G*. *paganellus*. Additionally, the OC rates of chemically induced predation stress were investigated and compared towards induced increased OC rates after handling and visual disturbance.

The current study was undertaken under the supervision of an accredited expert in laboratory animal science by the Portuguese Veterinary Authority (DGV-Portugal, following FELASA category C recommendations), according to the guidelines on the protection of animals used for scientific purposes from the European directive 2010/63/UE, and approved by the Órgão Responsável pelo Bem-Estar dos Animais (ORBEA) from the Polytechnic of Leiria. The collection of the organisms was conducted by Flying Sharks–Consultoria e Inovação Lda., based in Horta (Azores, Portugal), and fully licenced by www.dgav.pt (Veterinarian Authority), www.dgrm.mm.gov.pt (Marine Resources Authority), and www.icnf.pt (Nature Conservation Authority).

### Target organism

Twenty-three individuals of *G*. *paganellus* were collected in rock pools during low tide at Gambôa Beach, Peniche, Portugal (N 39° 21' 53'', W 9° 22' 23''), using hand nets. Fish were then transported to the laboratory and maintained in a 1000 L recirculating system with a plastic tank filled with 600 L of natural seawater for two months to acclimate from transport and handling effects (photoperiod 12h:12h L:D, abrupt light transition from room top fluorescent tube lights, light intensity: approx. 430 lx; temperature 20 ± 1°C; salinity 32 ± 1; nitrite < 0.1 ppm; ammonia 0 ppm). PVC tubes and terracotta pots on the bottom of the tank were used to provide shelter. Prior to the respiration measurements, thirteen *G*. *paganellus* (mean SL 83 ± 5 mm) were transferred to a 200 L recirculating system providing the same conditions as described above. Each fish was individually placed in a perforated plastic compartment (16 x 16 x 25 cm) inside a rectangular plastic tank (95 x 55 x 50 cm) to standardize handling procedures prior to respiration measurements. PVC tubes (10 cm length, 5 cm in diameter) on the bottom of the compartments were used to provide shelter. Fish were further acclimated for seven days and fed with mussels (*Mytilus* sp.) every other day, but starved 24 h prior to respirometry to exclude any effects on SMR [40]. The remaining ten individuals (mean SL 37 ± 10 mm) were used for the preparation of the predation stimuli.

### Predation stimuli preparation

#### Model predator

Common octopus (*Octopus vulgaris*) was chosen for predation stimulus preparation, as it is a top predator in rock pools. An adult octopus was kept in a 600 L plastic tank under the same maintenance conditions as for *G*. *paganellus*. PVC tubes on the bottom of the tank were used to provide shelter. The octopus was fed daily with prior frozen shrimps (Penaeidae) and mussels (*Mytilus* sp.).

#### Conspecific damaged-released alarm cues

Chemical alarm cues from conspecifics were prepared following the protocol from Larson & McCormick (2005) and McCormick & Manassa (2008) [14,28], with minor modifications. Specimens of *G*. *paganellus* (37 ± 10 mm SL, n = 10 in total) were euthanized by spinal cut and placed in a clean plastic petri dish. At each flank, 20 cuts of the skin with minor flesh damage were made with a clean razor blade. The fish was then rinsed in 100 mL of seawater, and was frozen afterwards at– 20°C for further usage. Skin extracts were prepared freshly for every respirometry trial to avoid potential loss of efficacy during storage or freezing [26].

#### Kairomones and dietary cues

To obtain the cues, the octopus was placed in a tank filled with 40 L natural seawater with continuous aeration for 4 h in total. The octopus was fed with two prior frozen *G*. *paganellus* that were used for the preparation of skin extract before, and after this 4 h period, the octopus was removed from the tank. The procedure was always conducted at the same time of the day (09:00 a.m.– 13.00 p.m.). Exact concentrations of predator cues cannot be provided, as the chemical components that induce the antipredator responses are still not identified. Therefore, for standardization purposes, exposure concentration of cues was set as the number of predators per hour per litre. The treatment water (0.0065 octopus h^-1^ L^-1^) was prepared freshly for every respirometry trial following the same routine and used immediately to avoid potential loss of efficacy during storage or freezing [26].

#### Predation simulation treatment water

The fresh skin extract from the two fish (2 x 100 mL) was properly mixed with the 40 L containing the predator kairomones and dietary cues. Due to increased ammonia levels, the predation treatment water had to be diluted. Preliminary tests defined a 1:10 dilution to achieve ammonia levels below 0.25 ppm. To remove any solids, the mixture was filtered (50 μm) before usage. For standardization purposes and avoidance of declining efficacy, the addition of the treatment water to the respiratory setup took place within 30 min after preparation.

### Experimental design

**Fig 1:**
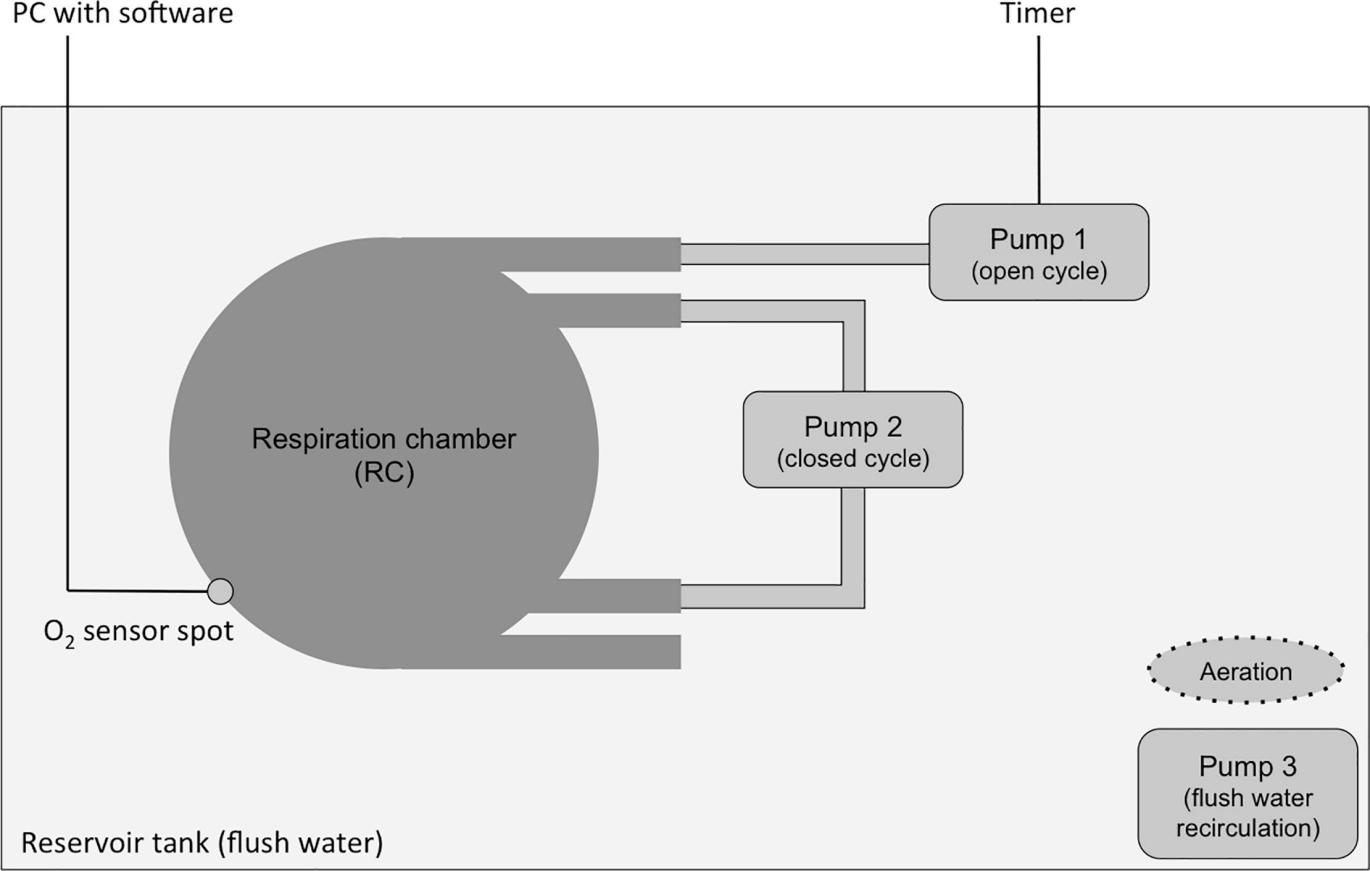
Schematic representation of the experimental setup. The oxygen concentration within the respiration chamber (RC) was continuously monitored using optical fibres connected to oxygen sensor spots. Pump 1 flushed the RC with aerated reservoir water in constant time intervals controlled by a timer. Pump 2 provided constant water circulation within the RC during the whole experimental period. Up to four RC were placed simultaneously into the reservoir tank. Pump 3 ensured water circulation in the reservoir tank to provide similar flush conditions for all RC. The reservoir tank provided space for maximum four RC in parallel. Modified after [41].

**Fig 2:**
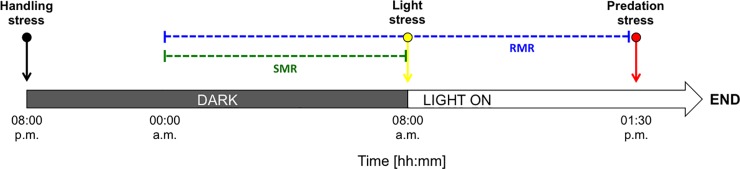
Schematic representation of the time setup of one experimental run. SMR: Standard metabolic rate (green dashed line). RMR: Routine metabolic rate (blue dashed line). The arrows indicate the time of the respiratory cycles that were used for assessing handling stress response (black; 08:00 p.m.), light stress response (yellow; 08:00 a.m.), and predation stress response (red; 01:30 p.m.). The time spans used for assessing SMR (00:00 a.m.– 08:00 a.m.) and RMR (00:00 a.m.– 01:30 p.m.) are given in dashed lines (green: SMR; blue: RMR). The experimental runs were conducted on five consecutive days to finally reach n = 13.

Fig 1 provides a schematic representation of the respiratory measurements that were conducted following the experimental setup from Kegler et al. (2015) [41]. Each fish was placed individually into a gas-tight acrylic respiration chamber (RC; Volume 0.5 L) (JeMiTec, Germany), and one RC remained empty and served as control. Throughout all measurements, there was no bacterial respiration detected. The RCs were placed into a 200 L reservoir tank filled with aerated, filtered (0.8/0.2 μm; PALL Corporation; USA) natural seawater. As the reservoir tank provided space for maximum four RCs in parallel, five experimental runs (see Fig 2) were conducted on consecutive days to finally reach thirteen replicates. Each individual fish was used only once in the experiments.

The time interval of one respirometry cycle was 45 min and consisted of three phases: flush (15 min), wait (5 min), and measurement (15 min). An analogue timer automatically controlled a small pump (Eheim, compact 300, Germany) that flushed the RC with aerated reservoir water (> 95% saturation within the RC). A second pump (Eheim, Aquabee UP 300, Germany) continuously circulated the water inside the RC through an external loop of gas tight Tygon tubing (Tygon® r3603, Saint-Gobain Performance Plastics Verneret/Charny, France). A third pump (Eheim, Aquabee UP 300, Germany) ensured water circulation in the reservoir tank to provide similar reservoir water conditions for all RCs. The decline of oxygen concentration within the closed RC was recorded using a Firesting-system (Pyroscience GmbH, Germany), consisting of optical fibres connected to sensor spots. Data were directly logged with the Pyroscience software (time interval 1 sec). The oxygen consumption (OC) rate was determined by adding a linear trend line over the 15 min measurement period and standardized to the OC per h. Exact RC water volume, wet weight (WW; to the nearest mg), total length (TL; to the nearest mm), and standard length (SL; to the nearest mm) for each fish were further determined after the experiment to calculate standardized OC rates in mg O_2_ h^-1^ g^-1^ WW.

#### RMR and SMR assessment

The respiration measurements followed a fixed time protocol (see Fig 2). All experimental runs started at 08:00 p.m. and light was switched off. For acclimation purposes, respiratory cycles from 08:00 p.m. until midnight were excluded from the estimation of SMR and RMR to avoid any confounding effects due to the handling stress treatment. At 08:00 a.m. the next day, light was switched on again. Respiratory cycles from midnight until daylight (08:00 a.m.) were used for best SMR estimation. For RMR calculations, respiratory cycles from midnight until the introduction of predation treatment water were included, considering both night and day periods. Slopes with R-squared values ≥ 0.82 were considered for assessing single average values of SMR and RMR for each fish, and standardized SMR and RMR were given in mg O_2_ h^-1^ g^-1^ WW.

#### Oxygen consumption rate at handling, visual disturbance, and predation risk

Handling stress was directly addressed at the beginning of the experiment when the fish was placed into the RC. Briefly, the fish was taken out of the single compartment in the maintenance tank using a soft-meshed hand net followed by a 10 sec air exposure, before being placed into the RC. The housing of the fish in the single compartments allowed for a standardized routine of taking the fish out and similar handling treatment for each fish. The first respiratory cycle after placing the fish in the RC (08:00 p.m.; excluded in SMR and RMR assessment, see Fig 2) was used to assess OC rate after handling stress. Visual disturbance was generated via the abrupt change from dark to light conditions in the morning (08:00 a.m.). Preliminary tests prior to the experiments proved enhanced OC rates of the fish inside the RC due to abrupt change from dark to light. The first respiratory daylight cycle (see Fig 2) was therefore used to represent OC rate after visual disturbance, respectively. Predation stress was chemically induced using the prepared predation treatment water. The treatment water was membrane-filtered (0.8/0.2 μm; PALL Corporation; USA) to minimize bacterial content, and pumped through an external flexible plastic tube into the reservoir tank. The tube was submerged so that no water surface disturbances induce fish reactions due to external disturbances. A preliminary test with soluble dye proved that added water took 2 min to be evenly distributed inside the reservoir tank. Therefore, predation treatment water was added during a closed respirometry cycle to ensure that all RC were flushed with similar water conditions, independent of their locations in the reservoir tank. Nine L of predation treatment water were added within 10 min at approx. 01:30 p.m. ± 20 min, depending on the closed respiratory cycles. For assessing the respiratory response to predation simulation, the OC rate was monitored within the first closed respiratory cycle after adding the predation treatment water (Fig 2).

During the whole experimental period, fish were not disturbed by other visual or audible stimuli to ensure that fish reaction was not provoked by surrounding noise rather than the induced stressors. OC rates were standardized and given in mg O_2_ h^-1^ g^-1^ WW.

### Statistical analysis

Oxygen consumption rates were calculated using Microsoft Excel 2011. Individual SMR and RMR values were determined taking averages from 7–12 and 15–21 respiratory cycles, respectively. Results are given as mean values ± standard deviation (SD). Data were tested for normality and homoscedasticity, and since assumptions of normality and homoscedasticity were not met, statistical differences were tested with the non-parametric Kruskal-Wallis test, followed by the Games-Howell post-hoc test for multiple comparisons of group means [42]. For all statistical tests, the significance level was set at *P* ≤ 0.05. Graphics were created with SigmaPlot software v11.0 (Systat Software Inc.) and the statistical analyses were performed in IBM SPSS Statistics 22.

## Results

Table 1 shows the results for mass corrected SMR and RMR, as well as biometric data for each individual.

**Table 1:** Standard and routine metabolic rates of *Gobius paganellus*. Biometric data are given for each individual (A-N).

.Fish ID	A	B	C	E	F	G	H	I	J	K	L	M	N
**WW** [g]	13.77	14.11	17.70	14.11	11.56	13.52	14.75	14.74	12.09	10.34	11.27	10.97	9.40
**TL** [cm]	10.4	10.5	11.3	10.4	10.0	10.3	10.4	10.5	9.7	9.4	9.8	9.7	9.4
**SMR** [mg h^-1^ g^-1^]	0.023	0.031	0.032	0.029	0.028	0.040	0.023	0.032	0.020	0.032	0.025	0.051	0.025
**RMR** [mg h^-1^ g^-1^]	0.033	0.051	0.041	0.041	0.036	0.045	0.032	0.044	0.037	0.039	0.038	0.060	0.035

WW: Wet weight; TL: Total length; SMR: Standard metabolic rate; RMR: Routine metabolic rate; n = 13

**Fig 3:**
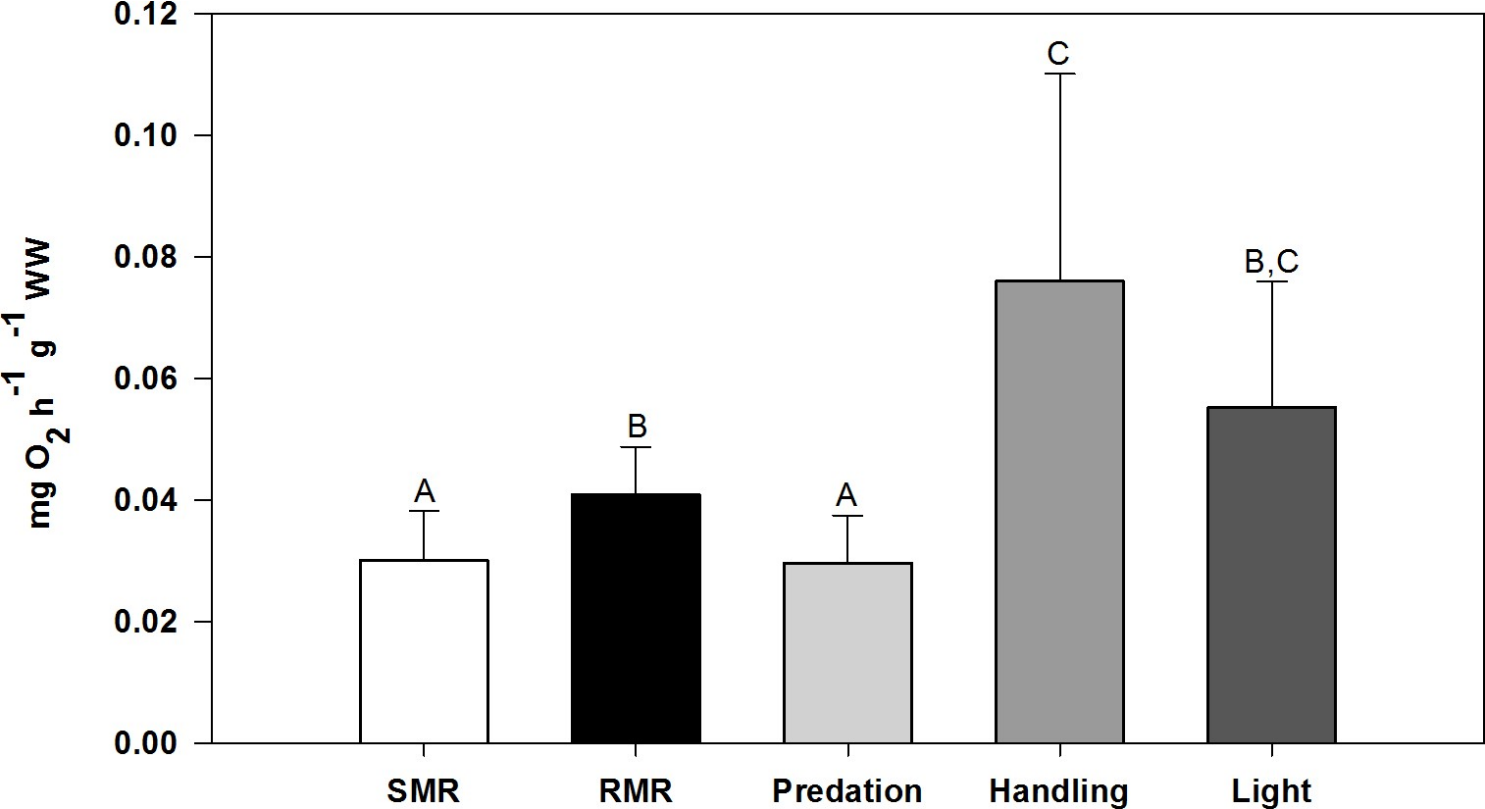
Oxygen consumption (OC) rates (mg O_2_ h^-1^ g^-1^ WW) of the rock goby *Gobius paganellus* under different stressors. Results are given in mean values ± SD. SMR: Standard metabolic rate. RMR: Routine metabolic rate. Predation: OC rate after introduction of predation treatment water. Handling: OC rate after handling. Light: OC rate after abrupt change from dark to light. Different capital letters indicate statistically significant differences between treatment groups (Kruskal-Wallis, Games-Howell, significance level: *P* ≤ 0.05). (n = 13; Light treatment: n = 10).

For *G*. *paganellus* (n = 13), the mean SMR was 0.0301 ± 0.0081 mg O_2_ h^-1^ g^-1^ WW (mean ± SD), which was significantly lower than the mean RMR being 0.0409 ± 0.0078 mg O_2_ h^-1^ g^-1^ WW (mean ± SD; Games-Howell, *P* = 0.015). In comparison to SMR and RMR, OC rates with predation stress were not significantly different from SMR, but were significantly lower than RMR (Games-Howell, *P* = 0.009), handling stress (Games-Howell, *P* = 0.003), and light stress (Games-Howell, *P* = 0.023) (see Fig 3). OC rates at handling stress were significantly higher than RMR (Games-Howell, *P* = 0.021) and SMR (Games-Howell, *P* = 0.003).

## Discussion

### Predation risk simulation

The challenge of this study was to simulate predation threat to *G*. *paganellus*. Several previous studies used real-life predators or predator models to provide visual cues to their target organisms (e.g., [11,14,15]). However, in this study chemical alarm signals were used. Much research has been done regarding chemical signals in predator-prey interactions (e.g., reviewed by [12,17,18]). Some researchers reported antipredator responses in gobiid species towards conspecific extracts (e.g., [26,27]), even with cross-reactions to skin extracts between two different gobiid species [43]. According to older studies from Pfeiffer (1960; 1977), *G*. *paganellus* does not have a so-called “Schreckstoff” in skin extracts, and no alarm substance cells (so-called club cells), in the epidermis [44,45]. Also Smith (1992), who described the alarm signal system of gobies, did not detect any alarm responses of *G*. *paganellus* to chemical stimuli from injured conspecifics [46]. However, he also pointed out that knowledge about the goby alarm system is still scarce and needs further investigation.

Test measurements before the main experiments showed that *G*. *paganellus* reacted with changes in OC rates to conspecific skin extracts, kairomones, and dietary cues from octopus fed with *G*. *paganellus*. These findings led to the assumption that *G*. *paganellus* respond to damaged-released epidermal compounds from conspecifics. This observation is in contrast to the descriptions from Pfeiffer (1960; 1977) [44,45]. However, instead of club cells, large vacuolated cells were found in the epidermis of all examined gobies, in which some species store alarm cues that are released during mechanical injury (reviewed by [46]). In fact, epidermal tissues are generally the first parts to be damaged during an attack from a predator [18]. The chemical compounds that are released in case of skin damage are therefore reliably unique and most aquatic organisms respond with antipredator behaviour as epidermal damage on fish is mostly caused by actively foraging predators [18,19].

The aim of this work, however, was not to quantify predation risk or to determine which chemical cue caused the stress response. Therefore, to mimic a relevant predation risk to *Gobius paganellus*, predator kairomones, dietary cues, and conspecific skin extracts were combined [47,48], as the component of the predation chemicals that induces the OC response is still unknown. As all other parameters were kept constant and the pre-tests only differed in presence or absence of chemical predation cues, these results enabled the assumption that *G*. *paganellus* experienced some level of predation stress. The following assessment of OC rates ultimately confirmed the initial observations.

### Respiratory assessment

#### RMR and SMR assessment

Results showed that SMR in *G*. *paganellus* was significantly lower than RMR (Fig 3), as expected. SMR is defined as the “minimum metabolic rate of survival” [36], i.e. the metabolic rate that can be measured at resting, without any stress and activity, at normothermic conditions [49,50]. This is why SMR measurements with diurnal species are usually done during the night [49,51]. RMR is defined as the OC rate of a post-absorptive, undisturbed fish. RMR is therefore usually estimated during the day including random activity of a diurnally active fish, e.g. digestion and reproductive investment, among others [50,51]. However, the results indicated that, although significantly different, RMR of *G*. *paganellus* is not much higher than SMR. This can be explained with the mode of life of *G*. *paganellus*. The rock goby, like many other members of the Gobiidae family, is a rather sedentary, demersal fish [21]. Due to its lifestyle, it is not as active as pelagic fish, and this can explain the rather close RMR and SMR values [51]. Similar results were found for the sand goby (*Gobius minutus*), when Healey (1972) concluded that RMR represents the metabolism at a very low activity level due to the sedentary way of life of such fish [52]. Moreover, from the ecological point of view, RMR measurements provide a suitable alternative to SMR assessments when focusing on the minimal costs of living in the natural environment, because fish in natural conditions are rather forced to spend energy to e.g. maintain posture with little movements [51].

#### Oxygen consumption rate at handling, visual disturbance, and predation risk

Apart from SMR and RMR estimation, respiratory responses of *G*. *paganellus* to different stressors were investigated. The OC results clearly show that *G*. *paganellus* reacted differently to different stressors (Fig 3). Handling stress and visual disturbance led to significantly higher OC rates than SMR, and handling stress led to even significantly higher OC rates than RMR, as expected. These findings are in line with the common physiological reactions towards physical stress. Handling stress, for example, increased plasma cortisol concentrations in yearling Chinook salmon (*Oncorhynchus tshawytscha*) and even resulted in mortality during severe handling of 30 min duration [37]. Barton & Schreck (1987) further showed a 2-fold increase of OC rates in physically stressed juvenile steelhead (*Salmo gairdneri*) compared to unstressed conspecifics, with a positive correlation to elevated plasma cortisol concentrations [38].

In contrast to the other stressors, however, oxygen uptake at predation stress was significantly lower than at handling stress and visual disturbance (Fig 3). In fact, oxygen uptake was even significantly lower than RMR, i.e. in mean values rather levelling with SMR. Several species react to predators with specific defence mechanisms to reduce the risk of being preyed upon [53]. Nile tilapia (*Oreochromis niloticus*), for instance, increase ventilation rates when visually exposed to a predator [54], and hardhead catfish (*Arius felis*) reacts to injured conspecifics with increased activity [55]. Similar to handling stress for example, oxygen uptake is increased during hyperventilation being essential when an escape reaction is needed, and therefore indicative for a “fight-or-flight” response [54]. *G*. *paganellus*, however, showed a significant decrease in OC rate at predation stress. This can be explained with a behavioural pattern known as “freezing” [26]. At risk of predation, which can be assessed by both visual and chemical alarm cues, many fishes, especially from the group of Gobiidae, reduce their movement, foraging, and can even hold breath. Additionally to reduced movement, gobiid fishes also tend to show bobbing behaviour (i.e. raising and lowering their bodies) to warn conspecifics and even other related species [56]. Smith (1989) demonstrated this stereotypical alarm response including reduced movement and bobbing behaviour for the tropical goby (*Asterropteryx semipunctatus*) to conspecific skin extracts [26], whereas McCormick & Larson (2007) made the same observations for this species in natural field conditions, verifying the chemical alarm cue system in the natural environment [29]. Another example is given for the bumblebee goby (*Brachygobius sabanus*), which also reduces movements as a response to chemical cues from injured conspecifics [27]. From an ecological point of view, these alarm responses increase the gobies’ survival, because reductions of foraging and movement increase their chance that predators may not recognize them. Due to their benthic way of life, they cannot show an extensive fight-or-flight response towards predators like it was shown from the pelagic fathead minnows [13,15] and Nile tilapia [54]. Due to their environment, gobies in intertidal rock pools are restricted in area to avoid predation, at least during low tide. Reducing their movement to maintain hidden from predators between the gravel or below rocks is therefore likely to be their strategy when under predation risk.

However, chronic exposure to predation risk and on-going freezing behaviour can get challenging regarding energetic parameters, especially because energy uptake is reduced (e.g., [57]). Following the threat-sensitivity hypothesis from Helfman (1989), chronic exposure towards high risk is rather seldom in nature, but levels of low- and high-risk situations usually change continuously [11]. Indeed, several studies indicated that challenging situations especially appear when predation stress is combined with other stressors. One stressor, for example, is food deprivation; Brown & Cowan (2000) report a trade-off between the response to conspecific alarm pheromones and foraging opportunities when finescale dace (*Chrosomus neogaeus*) was deprived of food [58]. This foraging-predation avoidance trade-off was also shown by feed-deprived Brazilian catfish pintado (*Pseudoplatystoma coruscans*). The fish showed only dashing behaviour when exposed to conspecific skin extracts, but no longer freezing behaviour like their fed control group [59]. The balance between predator avoidance behaviour and energy uptake becomes even more apparent at additional hyperthermia. Recent studies showed that juvenile damselfish (*Pomacentrus chrysurus*) respond to predation risk with reduced foraging at ambient temperature, but no longer at low food levels and increased temperature [60]. Pink & Abrahams (2016) report similar findings when investigating the anti-predator response in fathead minnows [15].

The results of the present respiration measurements indicate that *G*. *paganellus* reacted to predation stress with the stereotype response of freezing behaviour. For a clear picture, behavioural observations could additionally be considered. However, Rehnberg & Schreck (1987) found that behavioural reactions (i.e. a fight-or-flight response) do not necessarily mean a physiological stress response [31], which definitely underlines the importance of combined stress response assessments. As one of the aims of this study was to identify potential physiological impairments due to predation stress, cellular stress responses regarding the energy metabolism need to be considered for further understanding the underlying reasons and effects of reduced oxygen uptake at predation risk. In fact, loss of energy, which is generally related to growth, reproduction, and behaviour, has a higher probability to impair population level endpoints and thus communities. However, reduced metabolic rate is also observed in fish in hypoxic environments. Metabolic rate suppression occurs in many fish species and enables them to survive hypoxia due to the fact that they suppress their metabolic rate below RMR or even SMR [50]. In the present findings, aerobic metabolic rate of *G*. *paganellus* was also reduced below RMR towards the level of SMR as a response to predation risk. Since OC rates can be directly linked to the energy metabolism in organisms (reviewed by [32]), the correlation of respiratory results with cellular metabolic activities can provide an overall insight into the organisms’ stress responses. Thus, the investigation of energetic biomarkers ought to provide further information about the underlying cellular processes.

## Conclusions

In this study the metabolic response of *Gobius paganellus* to chemical predator alarm cues was investigated, by measuring OC rates. The rock goby reacted with changes in the RMR to the combination of *Octopus vulgaris* kairomones, dietary cues, and conspecific skin extracts. In contrast to increased OC rates due to handling and light stress, the exposure to chemical predation cues induced a significant reduction in OC rates below RMR. This metabolic suppression was interpreted as a result of the stereotypical freezing behaviour as antipredator response of gobiid fish. The observed metabolic suppression may also be shown in cellular respiration and/or selected biomarkers and should be further addressed. Our results underline the importance of biotic interactions in stress assessments, especially in a challenging era of unprecedented global changes, where exotic species presence may also impact the established natural dynamics of populations. Predation as a biotic factor should therefore be included in more realistic scenarios when addressing stress impacts in tidal rock pool organisms. Since single stressor assessments are far from ecologically relevant scenarios, the ecological relevance of multiple biotic and/or abiotic stress factors–including predation–has to be seen on population levels.

## Supporting information

S1 Supporting Information Results of statistical analysis. Statistical differences were tested with the non-parametric Kruskal-Wallis test, followed by the Games-Howell post-hoc test for multiple comparisons of group means.
(PDF)
