# Supplementary material for: Chemical predator signals induce metabolic suppression in rock goby (Gobius paganellus)
Source: PLoS One. 2018 Dec 17;13(12):e0209286. doi: 10.1371/journal.pone.0209286 (PMC6296658; doi:10.1371/journal.pone.0209286)
Supplement: S1 Supporting Information — Statistical differences were tested with the non-parametric Kruskal-Wallis test, followed by the Games-Howell post-hoc test for multiple comparisons of group means. (PDF) [file pone.0209286.s001.pdf]

S1 Supporting Information. Results of statistical analysis. Statistical differences were tested with the non-parametric Kruskal-Wallis test, followed by the Games-Howell post-hoc test for multiple comparisons of group means.

Oneway

Descriptives

O2 consumption

	N	Mean	Std. Deviation	Std. Error	95% Confidence Interval for Mean		Minimum	Maximum
					Lower Bound	Upper Bound		
Handling	13	,0760	,03410	,00946	,0554	,0966	,02	,14
Light	10	,0553	,02068	,00654	,0405	,0701	,02	,09
Predation	13	,0297	,00775	,00215	,0250	,0344	,02	,04
RMR	13	,0409	,00780	,00216	,0362	,0456	,03	,06
SMR	13	,0301	,00813	,00225	,0252	,0350	,02	,05
Total	62	,0460	,02551	,00324	,0395	,0524	,02	,14

Test of Homogeneity of Variances

O2 consumption

Levene Statistic	df1	df2	Sig.
9,645	4	57	,000

As the requirement for homogeneity of variances is not met (p-value<0,05), it is necessary to use the non-parametric test to perform the analysis of variance.

Non-parametric Kruskal-Wallis Test

Hypothesis Test Summary

	Null Hypothesis	Test	Sig.	Decision
1	The distribution of O2 consumption is the same across categories of id_treat.	Independent-Samples Kruskal-Wallis Test	,000	Reject the null hypothesis.

Asymptotic significances are displayed. The significance level is ,05.

Games-Howell Post Hoc Tests

Multiple Comparisons

Dependent Variable: O2 consumption

Games-Howell

(I) id_treat		Mean Difference (I-J)	Std. Error	Sig.	95% Confidence Interval	
					Lower Bound	Upper Bound
Handling	Light	,02073	,01150	,399	-,0137	,0551
	Predation	,04633*	,00970	,003	,0159	,0768
	RMR	,03507*	,00970	,021	,0046	,0655
	SMR	,04592*	,00972	,003	,0154	,0764
Light	Handling	-,02073	,01150	,399	-,0551	,0137
	Predation	,02560*	,00688	,023	,0033	,0479
	RMR	,01434	,00689	,293	-,0079	,0366
	SMR	,02519*	,00692	,025	,0029	,0475
Predation	Handling	-,04633*	,00970	,003	-,0768	-,0159
	Light	-,02560*	,00688	,023	-,0479	-,0033
	RMR	-,01126*	,00305	,009	-,0202	-,0023
	SMR	-,00042	,00311	1,000	-,0096	,0088
RMR	Handling	-,03507*	,00970	,021	-,0655	-,0046
	Light	-,01434	,00689	,293	-,0366	,0079
	Predation	,01126*	,00305	,009	,0023	,0202
	SMR	,01085*	,00312	,015	,0016	,0201
SMR	Handling	-,04592*	,00972	,003	-,0764	-,0154
	Light	-,02519*	,00692	,025	-,0475	-,0029
	Predation	,00042	,00311	1,000	-,0088	,0096
	RMR	-,01085*	,00312	,015	-,0201	-,0016

*. The mean difference is significant at the 0.05 level.
